# Incidence and correlates of chronic anemia in people living with HIV Initiating ART: An exploratory five-year retrospective cohort of patients retained in care

**DOI:** 10.1371/journal.pgph.0006133

**Published:** 2026-03-31

**Authors:** Kingsley Kamvuma, Benson M. Hamooya, John Amos Mulemena, Sepiso K. Masenga, Annet Kirabo, Sody M. Munsaka

**Affiliations:** 1 HAND Research Group, Mulungush University, School of Medicine and Health Sciences, Department of Pathology and Microbiology, Livingstone campus, Livingstone, Zambia; 2 University of Zambia, School of Health Sciences, Department of Biomedical Sciences, Lusaka, Zambia; 3 HAND Research Group, Mulungush University, School of Medicine and Health Sciences, Department of Public Health, Livingstone campus, Livingstone, Zambia; 4 Department of Medicine, Vanderbilt University Medical Center, Nashville, Tennessee, United States of America; 5 Vanderbilt Center for Immunobiology, Vanderbilt Institute for Infection, Immunology and Inflammation, Vanderbilt University Medical Center, Nashville, Tennessee, United States of America; 6 Vanderbilt Institute for Global Health, Vanderbilt University Medical Center, Nashville, Tennessee, United States of America; University of Colorado Anschutz Medical Campus: University of Colorado - Anschutz Medical Campus, UNITED STATES OF AMERICA

## Abstract

Chronic anemia remains a common and clinically important complication among people living with HIV (PLWH) receiving antiretroviral therapy (ART), contributing to increased morbidity and mortality, particularly in persistent or severe cases. This study estimated the incidence and correlates of chronic anemia among PLWH initiating ART in Zambia. A five-year retrospective cohort study was conducted among 243 adults who initiated ART between February 2015 and December 2020. Chronic anemia was defined as hemoglobin levels <13 g/dL for men and <12 g/dL for women persisting for more than six months. Hemoglobin levels were assessed at ART initiation and at least every six months thereafter. Participants were stratified by anemia status at baseline and followed for up to 60 months. Kaplan–Meier analysis was used to estimate anemia-free survival, and Cox proportional hazards models were used to identify exploratory correlates. Over five years, 125 participants (51.4%; 95% CI: 45.0%–57.9%) developed chronic anemia, with an overall incidence rate of 10.3 per 100 person-years. Among participants without anemia at baseline, the incidence rate was 2.91 per 100 person-years, while those with baseline anemia had a higher incidence of 15.31 per 100 person-years. Those with anemia at baseline, female sex (AHR 2.28; 95% CI: 1.12–4.62), severe anemia (AHR 8.06; 95% CI: 2.84–22.92), moderate anemia (AHR 5.72; 95% CI: 2.51–13.03), mild anemia (AHR 5.29; 95% CI: 2.15–12.99), and microcytosis (AHR 5.19; 95% CI: 1.19–22.74) were significantly associated with chronic anemia. Among participants without baseline anemia, zidovudine-containing regimens were independently associated with chronic anemia (AHR 4.43; 95% CI: 1.02–19.34). Chronic anemia remains a substantial clinical problem among PLWH receiving ART, particularly among those with baseline anemia and specific hematologic risk factors. These findings highlight the importance of early identification and monitoring of individuals at risk of persistent anemia during ART.

## Introduction

In 2023, approximately 39.9 million people were living with HIV (PLWH) globally. Despite advancements in HIV management with effective combinational antiretroviral therapy (ART), PLWH frequently experience haematological abnormalities, including chronic anemia. This condition is associated with disease progression and increased mortality [1–3]. A recent meta-analysis by Woldegeorgis et al. (2024) shows that chronic anemia significantly increases mortality risk in PLWH, especially in resource-limited settings, despite early ART initiation [4]. Prevalence of anemia in PLWH is highest in sub-Saharan Africa (SSA) (58–70%) [5–9]

Chronic anemia is a significant predictor of mortality among PLWH, with multiple studies indicating its substantial impact. A meta-analysis of 63 observational studies found that anemia independently increased the risk of mortality in PLWH, with an adjusted hazard ratio (HR) of 1.38 [10]. In a Chinese cohort, anemia was linked to a 74% increase in death risk (AHR: 1.74) [11]. In Tanzania, chronic anemia after six months of ART was associated with a fourfold increase in mortality risk, particularly among women and low-income individuals [12]. Similarly, among PLWH with tuberculosis, chronic anemia during antitubercular therapy increased mortality and treatment failure risk by up to 3.8 times [13]. These findings emphasize the critical need for early detection and management of chronic anemia to improve survival outcomes in PLWH.

Anemia is often exacerbated before the initiation of ART, where the interplay between HIV infection, chronic inflammation and immune activation can influence its development [14]. Several studies have reported that ART reduces the prevalence of anemia, but some patients still experience unresolved chronic anemia [15–18]. People living with HIV who begin ART with anemia are more likely to experience complications such as diminished immune recovery, increased susceptibility to infections, and reduced overall survival [19,20]. Understanding the predictors and mechanisms of unsolved anemia in PLWH initiating ART is crucial for improving patient management and outcomes [21].

Despite progress in HIV care, unresolved chronic anemia remains a key challenge in PLWH, particularly in SSA where healthcare resources are often limited [22,23]. Mechanisms driving this on-going chronic anemia, especially among those starting ART, remain insufficiently defined [24]. Consequently, interventions to address anemia in this population are limited, hindering quality of care and health outcomes. While previous studies have primarily focused on anemia prevalence at a single time point, there is limited evidence on chronic anemia, defined as persistent anemia lasting many months, among people living with HIV. Understanding chronic anemia is critical, as its persistence may contribute to ongoing immune dysfunction, increased morbidity, and poorer long-term outcomes compared to transient or anemia measured at one time point. By investigating the incidence and predictors of chronic anemia in PLWH who are initiating ART, this study seeks to fill a critical gap in understanding, ultimately aiming to improve management strategies and patient quality of life [25]. This study investigated the incidence and correlates of chronic anemia in PLWH initiating ART.

## Methods

### Study site and design

We conducted a retrospective cohort study on PLWH from Livingstone Teaching Hospital (LUTH), in Livingstone Zambia. This is a city located in the southern part of Zambia and is the tourist capital of Zambia [26,27]. Livingstone University Teaching Hospital provides HIV care and treatment services to almost 4000 PLWH annually. A retrospective follow-up study was conducted from 1 February 2015 to December 2020, among PLWH who attended ART clinic. We utilized programmatic data from the ART registry to identify 243 PLWH from Livingstone University Teaching Hospital in Zambia.

Hemoglobin levels were assessed at baseline (upon ART initiation), at six months, and then annually as part of routine clinical monitoring, with more frequent measurements for patients identified as anemic according to clinical guidelines.. Participants were followed for up to 60 months; however, not all individuals remained in the study for the full duration. Some participants were censored due to loss to follow-up, while others reached the study outcome (chronic anemia) before the end of the follow-up period.

### Study participants

Participants were eligible if they were HIV-positive adults (≥18 years) who initiated ART at LUTH between 1 February 2015 and 31 December 2020 and had documented baseline clinical and laboratory data at ART initiation. Approximately 4,000 PLWH receive care at LUTH annually, indicating the total number of PLWH receiving care not the number of participant eligible for the study. During the study period, 375 individuals initiated ART at LUTH and had retrievable baseline records, forming the source population for this analysis.

**Fig 1:**
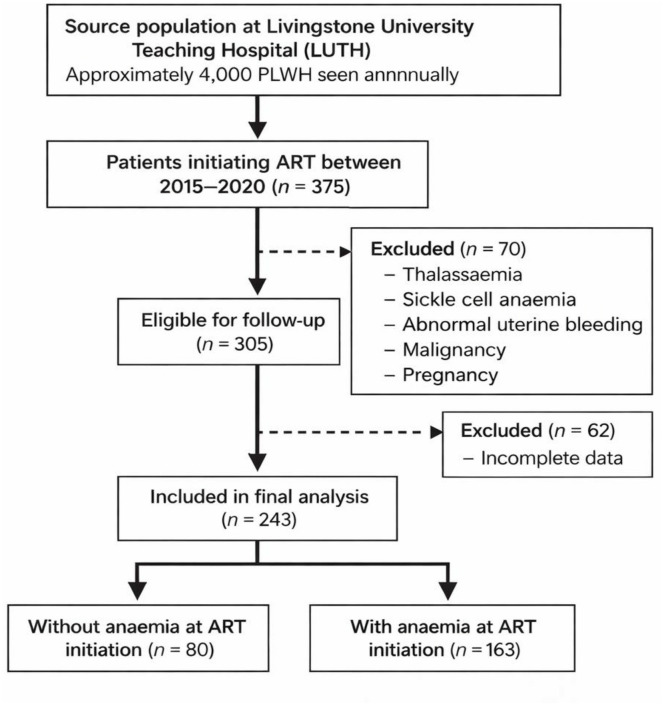
Flow chart for inclusion and exclusion criteria. Created with biorender.com.

We excluded individuals with a documented history of blood disorders (including sickle cell disease, thalassemia, or abnormal uterine bleeding), those who were pregnant at baseline, or those with a diagnosed malignancy. Participants with incomplete baseline clinical data or insufficient follow-up information were also excluded. For the longitudinal analysis of chronic anemia, we further restricted the cohort to individuals who remained enrolled in care and had hemoglobin measurements available during follow-up, to allow ascertainment of anemia persistence over time. After applying all exclusion criteria, 243 participants were included in the final analytic cohort (Fig 1). Because inclusion required longitudinal and sufficient follow up data, the analytic cohort likely represents more stable and adherent patients. Individuals lost to follow-up, transferred out, or with incomplete records may have had different risk profiles. Therefore, incidence estimates should be interpreted as minimum estimates, and observed associations may differ in the broader population of PLWH initiating ART.

### Data collection

Baseline data were extracted from medical records and included demographic characteristics (age, sex, weight, height), clinical parameters (hemoglobin level, mean cell volume (MCV), CD4 + cell count, viral load, white blood cell count, ALT, AST, creatinine, urea, platelet count), ART regimens, and follow-up data on anemia status and time to the defined diagnosis of chronic anemia.

We reviewed data for PLWH who enrolled in the ART clinic between 2015 and 2020, focusing on files with complete information that met our eligibility criteria. As is common with secondary data, we encountered substantial missing participants’ information. Our inclusion criteria limited the analysis to individuals who remained enrolled in the ART clinic for the follow up period. Data was abstracted from the SMART CARE database an electronic data management software for HIV patients, an electronic laboratory information system and patient files capturing sociodemographic and clinical characteristics at baseline and during follow-up. Follow-up time for each participant began at ART initiation, regardless of baseline anemia status. Individuals who were anemic at baseline contributed to the follow-up time from the same starting point as non-anemic individuals.

### Study variables

The variables were collected at baseline (the day of ART initiation) and on a five-year follow-up period. Most participants were initially started on non-nucleoside reverse transcriptase inhibitors (NNRTIs). However, some regimens were switched during treatment, transitioning mainly to an integrase strand transfer inhibitor (INSTI) while others remained on NNRTIs until the final follow-up period [21]. We further collected data on the type of ART regimen based on nucleoside reverse transcriptase inhibitor [NRTI] backbones: zidovudine [AZT], stavudine [D4T], tenofovir disoproxil fumarate [TDF], and abacavir [ABC]) to assess their risk for chronic anemia [28]. Furthermore, we estimated the glomerular filtration rate (eGFR) using the updated CKD-EPI 2021 equation, which estimates kidney function based on serum creatinine, age, and sex [29]. CD4 + counts were categorized as ≥351, 200–350, or <200 cells/mm³ [3].

Hemoglobin levels were recorded at baseline, six months, and annually throughout the follow-up period. MCV was categorized based on clinical thresholds for anemia classification, while other variables were analyzed continuously for more precise associations with chronic anemia. Information on alcohol use and smoking was not systematically recorded in the patient medical records and was therefore unavailable for analysis in this study. While these factors are known to influence anemia risk, our analysis focused on variables for which complete data were available. All covariates were selected based on the review of literature and clinical expertise.

### Operational definitions

Anemia was defined as haemoglobin <13 g/dL for men and <12 g/dL for women, according to the World Health Organization criteria [30]. Subclassified the anemia as mild (11–12.9 g/dl for males and 11–11.9 g/dl for females), moderate (8–10.9/dl), and severe (<7.9 g/dl) [30]. The type of anemia was assessed using mean cell volume (MCV) values, categorizing them as microcytosis (<80 fL), normocytosis (80–100 fL), and macrocytosis (>100 fL) [31]. The primary outcome Chronic anemia was defined as anemia persisting for at least 6 months, identified through hemoglobin laboratory results taken on two separate occasions at least 6 months apart, with consistently low levels in the anaemic range, i.e., < 13 g/dL for men and <12 g/dL for women during the follow up period as previously defined in another study [3].

### Statistical analysis

We used the statistical package for Social Sciences version 22 (SPSS) software for data analysis. Chi-square was used for categorical characteristics by chronic anemia status. Shapiro Wilk test was used to test for the normality of the data. Mann-Whitney was used to compare medians of two continuous variables if the data was not normally distributed. Chronic anemia-free survival time was compared based on sex, anemia status at baseline, and MCV values at baseline using Kaplan-Meier curves. Univariable and multivariable Cox proportional hazards regression were employed to assess the contribution of various factors in predicting the time to chronic anemia diagnosis while adjusting for potential confounders. Missing data were assessed using Little’s MCAR test, which rejected the assumption that data were missing completely at random. Patterns of missingness suggested potential missing not at random (MNAR) mechanisms. Given the extent and structure of missingness, a complete-case analysis approach was used. We acknowledge that under MNAR conditions, complete-case analysis may yield biased estimates. Clinically relevant factors identified in prior studies were included to avoid omitted-variable bias. Statistical significance was assessed using 95% confidence interval (CI) and 5% level of significance.

### Ethical approval

This study was approved by the University of Zambia Biomedical Research Ethics Committee (UNZABREC- REF. NO. 4062–2023). The study was conducted by the Declaration of Helsinki and Good Clinical Practice guidelines. Ethical considerations included maintaining participant confidentiality, ensuring the secure handling of patient data, and minimizing the risk of identification by ensuring that the data were de-identified. Data was collected between February - May 2024 at Livingstone University Teaching Hospital (LUTH).

## Results

### Participants characteristic

**Fig 2:**
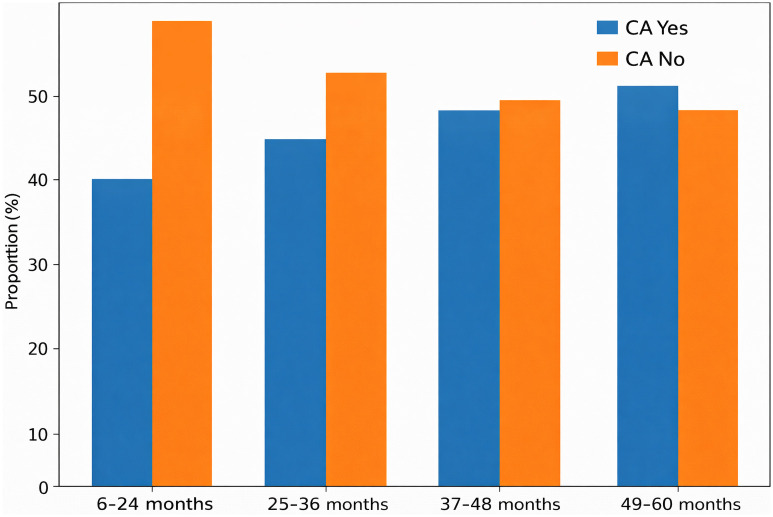
Cumulative proportion of PLWH developing chronic anemia (CA) from baseline to 60 months.

243 PLWH were included in these analyses (Table 1). Baseline characteristics for all study participants, as well as characteristics for the 125 participants who developed chronic anemia during follow-up (Table 1). For the overall population, the median age of study participants was 42.0 (IQR 32–51) years at baseline, with a 66% female predominance. Over 60-month follow-up period, the proportion of participants who developed chronic anemia increased steadily (Fig 2). Initially, 40% developed chronic anemia within the first 24 months, rising to 51.4% by the end of the study period (p < 0.001). Participants with baseline anemia had a high risk of chronic anemia, with only 31.9% recovering from anemia by Year 5, indicating that they were likely to remain anemic and develop chronic anemia. In contrast, non-anemic participants were less likely to develop chronic anemia, with over 80% maintaining their status throughout follow-up (Table 1).

**Table 1:** Proportion of Participants Remaining Free of Chronic Anemia Over Five Years Among PLWH on ART.

Follow up time	Initiation	Year 1	Year 2	Year 3	Year 4	Year 5
**Anaemic at baseline (n,%)**	163 (100)	154 (94.5)	66 (40.5)	62 (38.0)	54 (33.1)	52 (31.9)
**Non anemia at baseline (n,%)**	80 (100)	80 (100)	80 (100)	67 (83.8)	65 (81.3)	65 (81.3)

The declining proportions and absolute numbers represent participants who remained free of chronic anemia at each follow-up time point, starting from 100% at baseline.

### Baseline characteristic and development of chronic anemia

The analysis of baseline characteristics revealed several significant differences between individuals who developed chronic anemia and those who did not (Table 1). We found a higher proportion of females (60.2% vs 30.6%) developing chronic anemia compared to males (p < 0.001). Participants who developed chronic anemia had a lower median weight when compared to those who did not (60 kg vs. 65 kg, p = 0.017). Additionally, the severity of anemia at initiation was markedly higher in those who developed chronic anemia, with severe anemia present in 89.5% of this group compared to 20% in the non-chronic anemia group (p < 0.001). The Mean Cell Volume (MCV) was significantly reduced in individuals with chronic anemia, with microcytosis present in 83.1% compared to 16.9% in the non-chronic anemia group (p < 0.001) (Table 2).

**Table 2:** Baseline Characteristics and Development of Chronic Anemia in PLWH.

Background Characteristics at Initiation	Total (n)	Developed Chronic anemia on follow up		*P*
		No (n)	Yes (n	
**Age,** *m, years* **(*IQR)***	243	40 (28,48)	29 (19, 43)	0.403
**SEX**				**<0.001**
*Male*	72	50 (69.4)	22 (30.6)	
*Female*	171	68 (39.8)	103 (60.2)	
**Height, *m*** *m (IQR)*	232	1.67 (1.6, 1.7)	1.65	0.390
**Weight,** *kg m (IQR)*	243	65 (53, 73)	60 (51, 66)	**0.017**
**BMI,** *kg/m2 m (IQR)*	232	22.0 (20.2, 25.8)	22.2 (19.4, 24.8)	0.642
**Systolic BP** *, mmHg m(IQR)*	194	129 (117.5, 139.8)	110 (98, 119.5)	0.313
*Missing*	49	–	–	
**Diastolic BP** *, mmHg m(IQR)*	194	75 (72.3, 103)	70 (67, 73)	0.546
*Missing*	49	–	–	
**ART Regimen**				0.72
*INSTI*	154	72 (46.9)	82 (53.1)	
*NNRTI*	70	35 (50)	35 (50)	
**NRTI Backbone**				**0.026**
*ABC*	16	13 (81.3)	3 (17.7)	
*AZT*	30	13 (43.3)	17 (56.7)	
*D4T*	33	12 (36.4)	21 (63.6)	
*TDF*	111	47 (42.3)	64 (57.6)	
**Anemia severity**				**<0.001**
*Severe*	19	2 (10.5)	17 (89.5)	
*Moderate*	73	15 (20.5)	58 (79.5)	
*Mild*	71	37 (52.1)	34 (47.9)	
*No anemia*	80	64 (80)	16 (20)	
**CD4 *Cells/mm*** ^***3***^				0.16
*≤200*	41	19 (46.3)	22 (53.7)	
*201-350*	35	20 (57.1)	15 (46.9)	
*≥351*	98	46(46.9)	52 (53.1)	
**Mean Cell Volume**				**<0.001**
*Microcytosis*	65	11 (16.9)	54 (83.1)	
*Normocytosis*	117	70 (59.8)	47 (40.2)	
*Macrocytosis*	29	21 (72.4)	8 (27.6)	
**Viral load,** *copies/L m (IQR)*	**191**	345 (24, 3329.8)	200 (20, 229)	0.367
*Missing*	**52**	–	–	
**WBC,** *cells/L m (IQR)*	243	3.7 (3.1, 4.4)	3.6 (1.9, 5.0)	**0.023**
**Urea,** *U/L m(IQR)*	177	3.3 (3.0, 4.2)	1.7 (1.6, 2.9)	0.361
*Missing*	66	–	–	
**Creatinine,** *µmol/L m(IQR)*	162	96.9 (76.8, 114.3)	67.8 (52.4, 73.9)	0.340
*Missing*	81	–	–	
**ALT,** *mmol/L mIQR*	174	24.9 (17.8, 39.2)	11.8 (8.5, 23.4)	**0.018**
*Missing*	69	–	–	
**AST,** *mmol/L m(IQR)*	175	27.1 (22.5, 49.9)	20.0 (15.3, 29.7)	0.564
*Missing*	68	–	–	
**Platelets,** *cells/L m(IQR)*	243	225 (187, 286)	281 (209, 314.5)	**0.024**
**GFR,** *ml/min m(IQR)*	162	88.1 (60, 99.9)	115.2 (56.5, 122.6)	0.214
*Missing*	81	–	–	

**Abbreviations**: ABC (Abacavir), ALT (Alanine aminotransferase), AST (Aspartate aminotransferase), AZT (Zidovudine), BMI (Body Mass Index), BP (Blood pressure), CD4 (Cluster of differentiation 4), D4T (Stavudine), GFR (Glomerular Filtration Rate), INSTI (Integrase Strand Transfer Inhibitor), m (media), n (total number), NNRTI (Non-Nucleoside Reverse Transcriptase Inhibitor), TDF (Tenofovir disoproxil fumarate), and WBC (White blood cells).

In terms of treatment regimens, individuals on the AZT an NRTI backbone had a higher likelihood of developing chronic anemia (56.7%) compared to those on the ABC backbone (17.7%, p = 0.026). Chronic anemia group had a lower median white cell count (3.6 x10^9/L) compared to the non-chronic anemia group (3.7 x10^9/L, p = 0.023). ALT levels were significantly lower in the chronic anemia group (median 11.8 U/L) compared to the non-chronic anemia group (median 24.9 U/L, p = 0.018). Additionally, platelet counts were higher in those who developed chronic anemia, with a median of 281 x10^9/L versus 225 x10^9/L in the non-chronic anemia group (p = 0.024) (Table 2).

### Incidence and time to develop chronic anemia during the follow-up period

**Fig 3:**
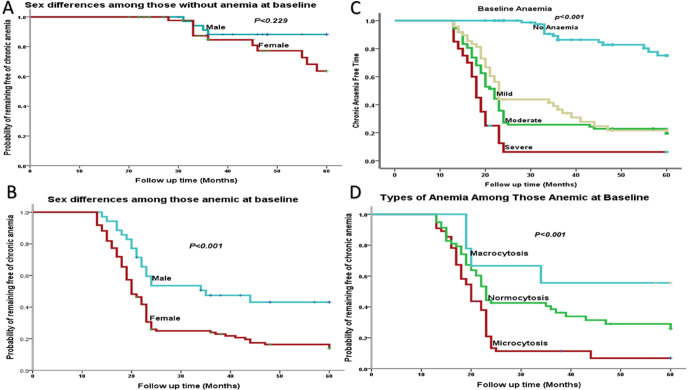
Kaplan–Meier curves illustrating the probability of developing chronic anemia over time. The cumulative incidence of chronic anemia was stratified by sex. Among participants who were non-anaemic at baseline, there was no significant difference between males and females (p = 0.289) **(A)**. However, among those who were anaemic at baseline, the incidence differed significantly by sex (p < 0.001) **(B)**. Cumulative incidence stratified by baseline anemia (C) status and MCV categories (D) showed significant differences.

This study showed that 125 participants (51.4, 95%:CI: 45.0% to 57.9%) of the adults initiating ART developed chronic anaemic in 5 person-years of observations (PYO), with an overall incidence rate of 10.3 per 100 person-years during the follow-up period. Among the cohort of PLWH who were free of anemia at baseline, the incidence rate of chronic anemia was found to be 2.91 per 100 person-years. In contrast, for those who were anaemic at baseline, the incidence rate was significantly higher, at 15.31 per 100 person-years (Fig 3).

The Kaplan-Meier curve (Fig 3A) illustrates the cumulative incidence of chronic anemia among non anaemic participants at baseline, stratified by sex. While females had a slightly higher cumulative incidence than males over the follow-up period, found non statistically significant difference (p = 0.289). Cumulative incidence of chronic anemia in those who were anaemic at baseline, also stratified by sex (Fig 3B). Females experienced a significantly higher incidence of chronic anemia when compared to males (p < 0.001) in the anaemic group.

We also used the Kaplan-meier curves to show the cumulative incidence of chronic anemia based on the severity of baseline anemia (Fig 3C). Individuals with severe anemia at baseline have the highest cumulative incidence, followed by moderate and mild anemia, while those without anemia have the lowest incidence (p < 0.001). This indicates a clear gradient where the anemia and its severity at baseline correlates with the risk of developing chronic anemia compared to those without anemia. Cumulative incidence of chronic anemia in individuals who were anaemic at baseline, stratified by their mean corpuscular volume (MCV) levels (Fig 3D). The data reveals that those with microcytosis at baseline exhibit the highest incidence of chronic anemia, followed by individuals with normocytosis and macrocytosis (p < 0.001).

### Correlates of chronic anemia among PLWH initiating ART treatment

In the adjusted Cox regression model, the associations between chronic anemia and several variables remained significant. Female sex continued to show a higher risk, with an adjusted hazard ratio (AHR) of 2.28 (95% CI: 1.12–4.62, p = 0.023). Microcytosis remained a significant predictor, with an AHR of 5.19 (95% CI: 1.19–22.74, p = 0.029). Anemia severity at initiation continued to be strongly associated with chronic anemia, with severe anemia showing the highest risk (AHR 8.06, 95% CI: 2.84–22.92, p = 0.001), followed by moderate anemia (AHR 5.72, p < 0.001) and mild anemia (AHR 5.29, p < 0.001). The use of AZT at ART initiation showed an adjusted hazard ratio (AHR) of 1.21 (95% CI: 0.24–6.13, p = 0.82), indicating no significant association (Table 3). However, confidence intervals were wide, reflecting small subgroup sizes and substantial uncertainty regarding the true magnitude of these associations.

**Table 3:** Shows correlates of chronic anemia in PLWH initiating ART.

Variables	Unadjusted analysis				Adjusted analysis			
	HR	95% CI		*P*	AHR	95% CI		*p*
**Sex**								
*Male*	1				1			
*Female*	3.56	1.99	6.33	**<0.001**	2.28	1.12	4.62	**0.023**
**Age,** *years*	1.00	0.98	1.02	0.80	1.02	0.99	1.05	0.07
**Weight,** *kg*	0.99	0.97	1.00	0.08				
**BMI,** *kg/m2*	0.98	0.93	1.03	0.41	0.97	0.92	1.01	0.15
**Viral load,** *copies/ml*	1.00	1.00	1.00	0.58				
**SBP,** *mmH*	0.99	0.97	1.00	0.14				
**DBP,** *mmHg*	0.98	0.96	1.00	0.06				
**CD4 + Count**	1							
*Low < 200*	0.91	0.38	2.16	0.82				
*Moderate 201–350*	0.59	0.24	1.46	0.25				
*High >351*	0.37	0.14	1.01	0.05				
**Mean Cell Volume**								
*Macrocytosis (>100)*	1				1			
*Normocytosis (80–99)*	1.74	0.71	4.25	0.23	3.12	0.72	13.60	0.13
*Microcytosis (<79)*	12.89	4.55	36.49	**0.001**	5.19	1.19	22.74	**0.029**
**ART Regimen**								
*INSTI*	1							
*NNRTI*	0.81	0.49	1.33	0.40				
**NRTI Backbone (Initiation)**								
*ABC*	1				1			
*AZT*	6.07	1.457	25.3	**0.013**	1.21	0.24	6.13	0.82
*D4T*	8.75	2.09	36.49	**0.003**	0.41	0.07	2.32	0.32
*TDF*	4.92	1.36	17.85	**0.015**	0.92	0.19	4.57	0.92
**Anemia severity at Initiation**								
*No Anemia*	1				1			
*Mild*	7.95	4.34	14.59	**0.001**	5.29	2.15	12.99	**<0.001**
*Moderate*	9.55	5.39	16.89	**0.001**	5.72	2.51	13.03	**<0.001**
*Severe*	18.57	9.15	37.67	**0.001**	8.06	2.84	22.92	**<0.001**
**WBC,** *cells/L*	0.91	0.79	1.04	0.16				
**Platelets,** *cells/L*	1.004	1.00	1.01	**0.02**	1.00	0.99	1.00	0.20
**Creatinine,** *µmol/L*	0.99	0.97	1.00	0.08				
**GFR,** *mL/min/1.73 m²*	0.99	0.99	1.00	0.64				
**Urea,** *mmol/L*	1.01	0.96	1.05	0.78				
**AST,** *U/L*	1.00	0.99	1.01	0.48				

Abbreviation: ABC (Abacavir), ALT (Alanine aminotransferase), AST (Aspartate aminotransferase), AZT (Zidovudine), BMI (Body Mass Index), BP (Blood pressure), CD4 (Cluster of differentiation 4), D4T (Stavudine), GFR (Glomerular Filtration Rate), INSTI (Integrase Strand Transfer Inhibitor), NNRTI (Non-Nucleoside Reverse Transcriptase Inhibitor), TDF (Tenofovir disoproxil fumarate), and WBC (White blood cells).

### Correlates of chronic anemia among participants with anemia at baseline

After adjusting for other variables, the associations remained strong for several factors. Female sex continued to be a significant risk factor for chronic anemia, with an adjusted hazard ratio (AHR) of 2.33 (95% CI: 1.06–5.12, p = 0.035). Microcytosis was associated with chronic anemia (AHR: 4.45, 95% CI: 1.02–19.43, p = 0.047). Severe anemia at baseline also continued to predict chronic anemia (AHR: 2.58, 95% CI: 1.06–6.24, p = 0.036) (Table 4). However, small subgroup sizes resulted in wide confidence intervals for several exploratory variables, indicating imprecision in effect estimates.

**Table 4:** Correlates of chronic anemia among participants with anemia at baseline.

Variables	Unadjusted analysis			Adjusted analysis		
	HR	95% CI	*P*	AHR	95% CI	*p*
Age, *years (m)*	0.99	0.98-1.01	0.23	0.99	0.97-1.01	0.39
Sex						
*Male*		1			1	
*Female*	2.17	1.32-3.56	**0.002**	2.33	1.06-5.12	**0.035**
BMI, *kg/m2*	0.99	0.96 -1.03	0.81			
ART Regimen						
NNRTI		1				
INSTI	1.37	0.82-2.30	0.23			
SBP *mmHg*	0.99	0.98-1.01	0.50			
DBP *mmHg*	0.99	0.98-1.01	0.06	1.01	0.99-1.04	0.38
Mean Cell Volume						
*Macrocytosis*		1			1	
*Microcytosis*	3.79	1.36-10.58	**0.011**	4.45	1.02-19.43	**0.047**
*Normocytosis*	2.11	0.76-5.92	0.15	3.53	0.81-15.28	0.09
CD4 + Count	1.001	1.00-1.001	0.11			
Anemia severity						
*Mild*		1			1	
*Severe*	2.24	1.25-4.03	**0.007**	2.58	1.06-6.24	**0.036**
*Moderate*	1.24	0.81-1.88	0.32	1.76	0.88-3.52	0.11
WBC *10*^*9*^*/L*	0.99	0.9-1.10	0.93			
eGFR *min/mL*	0.99	0.99-1.00	0.39			
Creatinine *g/dL*	1.00	0.99-1.01	0.89			

Abbreviation: ART (Antiretroviral therapy), BMI (Body Mass Index), DBP (Diastolic Blood Pressure), eGFR (Estimated Glomerular Filtration Rate), MCV (Mean Cell Volume), m (Median), mmHg (Millimetres of mercury), SBP (Systolic Blood Pressure), and WBC (White Cell Count).

### Correlates of chronic anemia among participants without anemia at baseline

In the adjusted analysis, the only participants on an AZT-containing regimens had an increased risk of developing chronic anemia among those who were free of anemia at initiation, AHR 4.43 (95% CI: 1.02–19.34, p = 0.048) (Table 5). However, small subgroup sizes resulted in wide confidence interval for the variables, indicating imprecision in effect estimates.

**Table 5:** Correlates of chronic anemia among participants without anemia at baseline.

Variables	Unadjusted analysis			Adjusted analysis		
	HR	95% CI	*P*	AHR	95% CI	*P*
Age, *years (m)*	0.99	0.95-1.03	0.52	1.00	0.95-1.05	0.88
Sex						
*Male*						
*Female*	2.58	0.82-8.11	0.11	2.41	0.61-9.49	0.21
BMI, *kg/m2*	0.95	0.88-1.02	0.18			
ART Regimen						
*NNRTI*						
*INSTI*	1.28	0.28-5.97	0.75			
SBP *mmHg*	1.01	0.98-1.04	0.70			
DBP *mmHg*	1.00	0.95-1.05	1.00			
Mean Cell Volume						
Macrocytosis						
Micro	2.11	0.53-8.51	0.29	1.97	0.37-10.58	0.43
Normo	0.78	0.23-2.65	0.69	1.07	0.24-4.76	0.93
CD4 + Count	1.001	0.99-1.00	0.48			
WBC, *10*^*9*^*/L*	0.8	0.61-1.29	0.536			
eGFR *min/mL*	1.01	1.00-1.02	0.08			
Creatinine *g/dL*	0.98	0.96-1.00	0.09			
NRTI Backbone						
*TDF*		1				
*ABC*	3.25	0.65-16.17	0.15	2.55	0.49-13.02	0.26
*AZT*	3.91	0.97-15.67	0.05	4.43	1.02-19.34	**0.048**
*D4T*	1.73	0.18-16.75	0.64	3.49	0.30-40.53	0.32

Abbreviations: ABC (Abacavir), ALT (Alanine aminotransferase), ART (Antiretroviral therapy), AST (Aspartate aminotransferase), AZT (Zidovudine), BMI (Body Mass Index), DBP (Diastolic Blood Pressure), D4T (Stavudine), eGFR (Estimated Glomerular Filtration Rate), GFR (Glomerular Filtration Rate), INSTI (Integrase Strand Transfer Inhibitor), MCV (Mean Cell Volume), m (Median), mmHg (Millimetres of mercury), NNRTI (Non-Nucleoside Reverse Transcriptase Inhibitor), SBP (Systolic Blood Pressure), TDF (Tenofovir disoproxil fumarate), and WBC (White Cell Count).

### Missing data assessment

To evaluate the potential impact of missing data, we compared baseline characteristics between participants included in the complete-case analysis and those with missing data (Table 6). Significant differences in viral load and ART regimen suggest that missingness may not have occurred at random, indicating potential bias in complete-case estimates.

**Table 6:** Baseline characteristics by missing data status.

Variable	Complete cases (n, %)	Missing data (n, %)	p-value
**Age** (mean ± SD)	44 (35.3- 51)	37.5 (29.3-49.8)	0.207
**Sex**			0.060
*Male*	61 (20.8)	16 (79.2	
*Female*	151 (88.3)	20 (10.7)	
**MCV**			0.177
*Microcytosis*	59 (90.8)	6 (9.2)	
*Normocytosis*	96 (81.4)	22 (18.6	
*Microcytosis*	26 (89.7)	3 (10.3)	
**CD4 *Cells/mm3***			0.130
*<200*	37 (90.2)	4 (9.8)	
*201-350*	29 (82.9)	6 (17.3	
*351-500*	19 (67.9)	9 (32.1)	
*>500*	32 (78)	9 (22)	
**NRTI Backborne**			0.078
ABC	11 (61.1)	7(39.9)	
*AZT*	3 (9.7)	28 (90.3)	
*D4T*	1 (3.0)	32 (97)	
*TDF*	20 (16.3)	103 (83.7)	
**ART Regimen**			0.02
*INSTI*	125 (79.1)	33 (10.1)	
*NNRTI*	40 (100)	0(0)	
**BMI *kg/m2 m (IQR)***	21.5 (19.5-25.3)	22.5 (19.5-25.2)	0.189
**Viral load *copies/L m (IQR)***	71.5 (20-847)	20 (0-45.8)	0.031
**SBP *kg/m2 m (IQR)***	122.5 (110.5-137)	120 (110-129.8)	0.830
**DBP *kg/m2 m (IQR)***	77.5 (70-93.3)	76 (68-85)	0.964
**MCV *fl m (IQR)***	89.9 (80-93.5)	85.6 (76.9-95.5)	0.059
**eGFR *µmol/L m(IQR)***	94.1 (74.6-114)	80.6 (64-96)	0.269
**Urea *U/L m(IQR)***	3.3 (2.4-3.8)	3.4 (2.9-4.3)	0.865

**Abbreviations**: ART, antiretroviral therapy; ABC, abacavir; AZT, zidovudine; D4T, stavudine; TDF, tenofovir disoproxil fumarate; INSTI, integrase strand transfer inhibitor; NNRTI, non-nucleoside reverse transcriptase inhibitor; BMI, body mass index; BP, blood pressure; MCV, mean corpuscular volume; eGFR, estimated glomerular filtration rate; IQR, interquartile range.

## Discussion

This study examined the incidence and exploratory associations of chronic anemia in a cohort of PLWH over a 5-year follow-up period after ART initiation. We found a substantial burden of chronic anemia in PLWH, with an overall incidence rate of 10.3 per 100 person-years. The incidence was higher in individuals who were anaemic at baseline (15.31 per 100 person-years) compared to those without anemia (2.91 per 100 person-years). These rates are lower than those reported in Zimbabwe (17.61 per 100 person-years) [17] and in the Ethiopian study (12 per 100 person-months of observations) [22], although those studies measured overall prevalent anemia rather than chronic anemia. Among individuals who were not anaemic at baseline, the incidence of chronic anemia in this study was higher than that reported in a U.S. study, which found an incidence of 0.46 per 100 person-years [3]. Differences in HIV care including better-resourced healthcare settings with routine monitoring, early ART initiation, and improved nutritional support may contribute to the marked disparities in incidence rates. These findings underscore the importance of implementing structured anemia assessment and management strategies that extend beyond achieving viral suppression alone. Remarkably, 68.1% of individuals who were anemic at baseline remained anemic during follow-up, indicating that anemia frequently persists despite ART initiation among patients retained in care. The high persistence observed in this cohort suggests that chronic anemia continues to represent a significant complication in the era of effective ART, even among individuals who maintain sustained viral suppression throughout follow-up [3,22,28].

Patients who were anaemic at the start of ART were more likely to either remain anaemic or develop chronic anemia over time. These findings emphasize that ART initiation does not always correct anemia, especially for individuals already anaemic at baseline. Variations exist depending on the population and setting however the development of chronic anemia was faster in certain subgroups, particularly females, those with severe anemia at baseline, and baseline low MCV values or a steeper decline in the probability of remaining free from chronic anemia.

The higher incidence of chronic anemia among females may be attributed to a combination of biological, hormonal, and socio-economic factors. Women generally have lower iron stores than men [18,32–34]. Menstrual blood loss further exacerbates this risk, especially in premenopausal women [35–37]. Additionally, higher rates of malnutrition and micronutrient deficiencies among females, particularly in resource-limited settings, may contribute to their increased vulnerability to chronic anemia [32]. Participants with anemia at baseline had a higher risk of developing chronic anemia compared to those without anemia at baseline and the risk was higher with more severe forms of anemia especially severe anemia. Baseline severe anemia was significantly associated with chronic anemia, likely reflecting underlying nutritional deficiencies, chronic inflammation, or bone marrow suppression [38,39].

Participants who were free of anemia at baseline had a much lower rate of developing chronic anemia. Several studies have reported that ART reduces the prevalence of anemia, but some patients still experience unresolved anemia [9,15,40]. Although ART is essential for managing HIV and can improve anemia, it is not always sufficient to correct existing anemia on its own, particularly since some ART regimens, like those containing zidovudine may even worsen the anemia [9,41]. Non-anaemic participants are also less likely to develop chronic anemia due to better immune function and fewer HIV-related commodities, whereas those with baseline anemia may face advanced disease and additional challenges like nutritional deficiencies [24].

Microcytosis or Low mean corpuscular volume (MCV) at baseline was associated with subsequent development of chronic anemia in this cohort. However, in the absence of iron studies, the underlying etiology of low MCV cannot be determined. Reduced MCV may reflect iron deficiency, thalassemia traits, anemia of chronic disease, or other hematologic conditions, particularly in settings with overlapping nutritional and genetic factors. Therefore, this finding should be interpreted as an observed association rather than evidence of a specific causal pathway. Further prospective studies incorporating iron parameters and hemoglobinopathy screening are required to clarify the mechanisms underlying this relationship.

Zidovudine’s well-known myelotoxic effects, particularly through its inhibition of mitochondrial DNA polymerase, impair erythropoiesis and cause bone marrow suppression, contributing to anemia over time [9,42,43]. This emphasizes the importance of transitioning away from AZT-based regimens, as recommended by the World Health Organization (WHO), which advocates for the use of less myelotoxic drugs [44].

This study has several important limitations. The analytic cohort consisted only of PLWH who initiated ART and were retained in care with adequate follow-up data, which likely introduced selection bias and limits generalizability to more stable patients; consequently, the reported incidence of chronic anemia may represent a conservative estimate. Missing data was a significant limitation of this study, key laboratory variables were not missing completely at random (MCAR), and patterns suggested potential missing not at random (MNAR) mechanisms. The use of complete-case analysis under these conditions may have introduced bias and affected the validity of effect estimates. Several associations were characterized by wide confidence intervals, reflecting small subgroup sizes and substantial uncertainty regarding the true magnitude of effects; therefore, multivariable models should be considered exploratory and hypothesis-generating. The retrospective design limited control over unmeasured confounders, including ART adherence, nutritional status, and iron-related parameters, and the absence of iron studies prevented determination of the etiology of low MCV values. Taken together, these limitations warrant cautious interpretation of the findings.

## Conclusion

In this selected cohort of PLWH retained in care, chronic anemia remained a common clinical problem. Among participants who were free of anemia at ART initiation, we observed an incidence rate of chronic anemia of 2.91 per 100 person-years, while those who were anemic at baseline experienced a substantially higher incidence rate of 15.31 per 100 person-years. Within this cohort, we observed associations between chronic anemia and baseline characteristics including female sex, severe anemia, and microcytosis, as well as Zidovudine-containing regimens among those without baseline anemia. Although some estimates were imprecise, the findings offer important insights into chronic anemia among PLWH and associated clinical correlates. These exploratory and hypothesis generating findings require confirmation in larger, prospective studies with comprehensive laboratory assessment and complete follow-up data. Nonetheless, the findings emphasize the need for anemia screening, nutritional assessment, and continued efforts to transition away from AZT-based regimens, particularly in resource-limited settings such as Zambia.

## Supporting information

S1 Data Raw data used for analysis. (XLS)

S1 STROBE Checklist A strobe checklist show where each reporting item is addressed in the manuscript*.* (PDF)
